# “SmartMonitor” — An Intelligent Security System for the Protection of Individuals and Small Properties with the Possibility of Home Automation

**DOI:** 10.3390/s140609922

**Published:** 2014-06-05

**Authors:** Dariusz Frejlichowski, Katarzyna Gościewska, Paweł Forczmański, Radosław Hofman

**Affiliations:** 1 Faculty of Computer Science and Information Technology, West Pomeranian University of Technology, Szczecin, Żołnierska 52, 71-210 Szczecin, Poland; E-Mails: kgosciewska@wi.zut.edu.pl (K.G.); pforczmanski@wi.zut.edu.pl (P.F.); 2 Smart Monitor sp. z o.o., Niemierzyńska 17a, 71-441 Szczecin, Poland; E-Mail: radekh@smartmonitor.pl

**Keywords:** “SmartMonitor”, visual surveillance, video content analysis, event detection, remote control, alarm, home automation

## Abstract

“SmartMonitor” is an intelligent security system based on image analysis that combines the advantages of alarm, video surveillance and home automation systems. The system is a complete solution that automatically reacts to every learned situation in a pre-specified way and has various applications, e.g., home and surrounding protection against unauthorized intrusion, crime detection or supervision over ill persons. The software is based on well-known and proven methods and algorithms for visual content analysis (VCA) that were appropriately modified and adopted to fit specific needs and create a video processing model which consists of foreground region detection and localization, candidate object extraction, object classification and tracking. In this paper, the “SmartMonitor” system is presented along with its architecture, employed methods and algorithms, and object analysis approach. Some experimental results on system operation are also provided. In the paper, focus is put on one of the aforementioned functionalities of the system, namely supervision over ill persons.

## 1. Introduction

“SmartMonitor” is an intelligent security system combining the advantages of an alarm system, video surveillance, visual content analysis (VCA) algorithms and home automation. The central unit of the system is built on the basis of electronic components specific to the personal computers (PCs) and supports cameras, sensors and controlled devices. The system is a complete solution and has various applications, e.g., protection against unauthorized intrusion, crime detection or supervision over ill persons. It can be utilized both inside and outside the building to increase security in houses, private apartments, nursing homes, small businesses or shops, as well as in backyards, gardens, parking spaces, building surroundings, especially near entrances or windows. “SmartMonitor” has already been described in literature [1–4].

There is a category of systems which has similar application to our supervision over ill persons scenario, such as ContextCare [5], Maisons Vill'Age [6] or a multi-sensor surveillance system described in [7]. These systems are intended to monitor ill or elderly people, however lack VCA functionality. The main tasks addressed by VCA are object detection, recognition and tracking. Many systems of such capabilities have been recently described in the literature, e.g., a system using dynamic saliency map for object detection and boosted Gaussian Mixture Model with Adaboosting algorithm for object classification [8], an automated surveillance system using omnidirectional camera and multiple object tracking [9], a smart alarm surveillance system using improved multiple frame motion detection and layered quantization compression technology for real-time surveillance with possibility of remote viewing [10] or a real-time surveillance system using integrated local texture patterns [11]. There are also some recent solutions combining both sensors and VCA. The authors of [12] presented an autonomous monitoring system for detecting environmental accidents, e.g., a fire based on color and flame behavior. The authors of [13] proposed an intelligent surveillance system for the identification of dangerous intrusions based on information from video, audio and sensors. The literature review indicates also the intelligent security systems based on different approaches. The authors of [14] proposed a system based on wireless sensor network for guarding important utilities without human intervention. Liao and Su [15] described an intelligent security system based on multiple communication interfaces, and intended for home automation. In [16] a modular security system for home automation is proposed. Each module has various interfaces and can be supervised directly or remotely.

The paper aims to present several aspects of the “SmartMonitor” system, especially the advantages of combining video content analysis algorithms, sensors and controlled devices. We provide the system characteristics and explain the approach which was implemented for the supervision of ill/elderly persons. The goal of the research described in the paper was to develop a video processing model for effective detection, classification and tracking of moving objects. The main implemented approaches included in the model are adaptive background modeling based on Gaussian Mixture Models and various color models, Haar and Histogram of Oriented Gradients (HOG) classifiers and Mean-Shift tracking.

The rest of the paper is organized as follows: the second section contains system characteristics and architecture. The third section discusses methods and algorithms employed, as well as some similar solutions that were considered. The fourth section describes the most important aspect of image analysis—the analysis of foreground objects, *i.e.*, moving objects and their features. The fifth section includes a description of experimental conditions and results. The last section concludes the paper.

## 2. System Characteristics and Architecture

### 2.1. System Characteristics

“SmartMonitor” has properties that are typical for traditional security solutions and monitoring systems, but expands them with the capabilities of intelligent video processing based on the identification of objects and events. This extends the functionality of the product by enabling moving object discrimination before the alarm activation, which is impossible when simple motion detectors are used. The system enables the design of individualized alarm responses which depend on the type of detected object or event. An alarm can be triggered by both the result of image analysis or a specified type of sensor, e.g., carbon monoxide detector, or a combination of various factors. A system response can be a single action or a set of actions, including a phone call, a siren, an email, a text message, a message played through a loudspeaker, a signal sent to the controlled device. The system offers remote access to several functionalities, such as activation or deactivation of the protection status, live preview of images from cameras or sending control signals to controlled devices to, for example, cut off the flow of current in a given socket. It should be highlighted that the system reacts automatically to every learned situation in a way that has been previously specified by the user.

The novelty of the system is expressed by the fact that we employ simple, yet effective methods that do not have high computational overhead, e.g., we join Haar-like features and Histogram of Oriented Gradients at the classification stage. Moreover, our solution is robust to environmental conditions and enables the use of low-cost cameras—low resolution images are enough to perform the surveillance. Furthermore, “SmartMonitor” belongs to the category of intelligent solutions. The intelligence of the system lies in the manner in which the system operates and in its ability to react automatically to the detected objects and events in a way pre-specified by a user. According to [17], the “intelligent video surveillance” refers to the efficient extraction of information from the video data by the automatic detection, tracking and recognition of objects of interest as well as the analysis and understanding of their activities and related events. All of the mentioned functionalities are offered by the “SmartMonitor” software and incorporated in its video processing model. The other aspect of the intelligence is the automatic and autonomic system response—it means that the system performs an action in response to an alarm, e.g., triggered by an object detected in the protected area, without any external interaction. This does not require a qualified employee for instant monitoring, reporting and action. Thanks to the use of wireless communication and controlled devices, “SmartMonitor” has the functionalities of home automation system, or in other words—it can be used for the management and control of the building and its nearest surrounding. This functionality may be activated directly or remotely by a user, or in a result of the non-alarm action—an exemplary rule: when a person gets up (in a certain room and hours) then turn on the kettle and rise the blinds. Therefore, the home automation using the “SmartMonitor” system has a twofold purpose: first, a user defines events or a schedule and assigns certain actions to them, which are performed automatically by the system, and second—a user connects to the system directly or remotely and issue the command to the system, e.g., cut off the current flow in a given socket. Examples are endless, depending mainly on the connected devices and user's needs.

To our knowledge, “SmartMonitor” is the first intelligent security system which combines the functionalities of different solutions, it is appropriate for home use and due to its price it is affordable for individual users. Intelligent monitoring systems that use VCA are often applied in public spaces, e.g., airports, or other areas which require a high level of protection. This is associated with the need for very specific, targeted and expensive infrastructure as well as the need to employ qualified employees who will respond to system alerts. For instance, IVA 5.60 (Intelligent Video Analysis) by Bosch is a guard assistant system based on intelligent video analysis. The system is able to detect, track and analyze moving objects. This functionalities are built into cameras and encoders, which increases the cost of installation. Bosch IVA is declared to be an industry solution [18,19]. AgentVi provides the another solution for video analysis, which is based on open architecture approach and designed especially for large installations. The software analyzing the video is distributed between an edge device (a camera or encoder) and a server [20]. These both solutions are undoubtedly based on advanced video analytics algorithms, however are not intended for home use. Their architectures also differs compared to the “SmartMonitor” which has centralized processing unit and does not process any data on the edge devices. Moreover, the mentioned systems do not enable the use of controlled devices. The other group of systems with similar characteristics include solutions like ADT Pulse [21] or vivint [22], that generate alerts based on various sensors. Their key features are: a use of wireless camera, home automation (e.g., door locks) and remote access. These solutions combine advantages of home security and automation just as our system, however require human intervention. Furthermore, they do not enable a differentiation of dangerous situations or automatic response. There are also some very advanced solutions available on the market which are meant for behavior recognition—one of them is AISight by BRS Labs [23]. This system autonomously builds a knowledge base and generates real-time alerts to support the security team in transportation, critical infrastructure, law enforcement and homeland security, and city and urban environments. It is able to analyze traffic, detect perimeter intrusion, secure facilities, generate transit and manufacturing alerts, and identifies various events and activities with respect to a usual time of a day. Compared to AISight, “SmartMonitor” system is not capable of self-learning, however, for the purposes of “SmartMonitor”, such functionality is redundant and might increase the final price. Moreover, this solution does not use controlled devices.

### 2.2. Logical and Physical Architecture

Logical and physical architectures reflect the system's shape. There are several essential and crucial elements which constitute a core of the system. The most important system components are: the multi-interface module, graphical user interface module, signal receivers, control modules/converters, encryption devices, identifying algorithm, repository of objects, contexts and patterns, recording module and the system for the connection to the environment.

The multi-interface module is responsible for two-way communication with peripheral devices. For example, it manages the sequence of messages to provide parameters required for each of the connected devices and ensure appropriate times of delivery and reliable delivery. The graphical user interface (GUI) module is responsible for generating images in the user interface. The interface can be operated via a web browser or mobile browser and offers multilingual features. Signal receivers include driver modules that support receiver streams, such as WiFi or Bluetooth, and other dedicated devices that receive analog signals. Control modules/converters support devices physically connected to the system, such as wired cameras or wired sensors. Encryption devices are used in analog or digital direct transmissions to protect transmitted data which are decoded in the multi-interface module. A crucial part in video processing stage is the identifying algorithm—it analyses images and reports detected parameters (e.g., an object's size or velocity) and event features (e.g., a fall). Moreover, it enables parallel processing of several video streams, while maintaining a relatively low use of computational resources and high reliability. The repository is responsible for combining parameters and features detected by the identifying algorithm into a definition of an object or event. The recording module is responsible for registration and appropriate storage of video streams and data related to the identified objects and events. The system for the connection to the environment is a secure interface, enabling communication between the system and Internet or mobile network.

**Figure 1.**
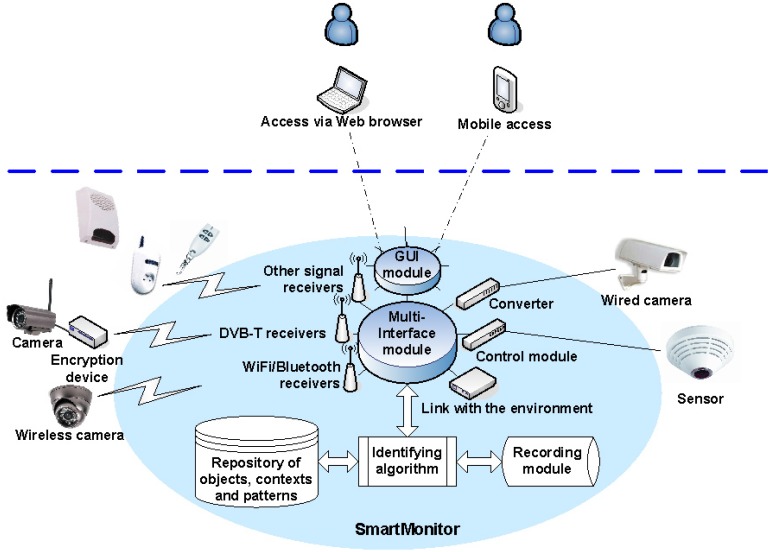
A simplified representation of the system core elements. The elements within light blue area are embedded in the central unit of the system.

The simplified general scheme of the core system elements is depicted in Figure 1. The light blue area reflects the elements embedded in the central unit of the system which is built of electronic components characteristic to PCs. The diversity of peripheral devices, both those sending data to the central unit and receiving signals from the system, enables the user to define complex system responses to a combination of many various factors. There are almost no restrictions on the choice of peripheral devices, such as cameras or sensors, however some fundamental requirements have to be met, e.g., good image quality or wireless transmission range. Emphasis was placed on the appropriate selection of electronic components responsible for wireless transmission or signal sending and receiving in order to support various peripherals. An example configuration of the “SmartMonitor” system is given below:

- Casing made of 1 mm thick metal sheet;
- Parameters of the PC-based components (central unit) are as follows: processor—Intel Core i5; memory—16 GB; hard drive—256 GB; ports—2× USB 3.0, 6× USB 2.0, 2× RJ45; 2 audio outputs; Bluetooth 3.0; 2 WiFi antennas; 2 antennas for control devices; operating system—64-bit Windows 7; power consumption—95 W;
- Additional equipment includes: Internet Protocol camera (1–4 pcs, Day and Night option required); Power over Ethernet (PoE) switch; additional Uninterruptible Power Supply (UPS); remotely controlled sockets and remote controllers.

### 2.3. Software

**Figure 2.**
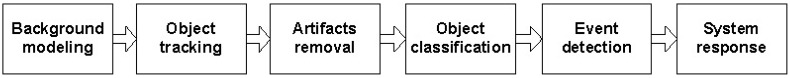
The simplified scheme of the main system modules.

The key part of the software concerns video content analysis, *i.e.*, an automatic analysis of video that aims mainly at detection, recognition and identification of objects and events present in the captured images. There are six main system modules responsible for VCA and other tasks associated with video processing and the interpretation of its results. Each module applies various methods and algorithms, but not all modules are active at a particular moment—the video data is transmitted and processed serially. The simplified scheme of the main system modules is depicted in Figure 2.

**Figure 3.**
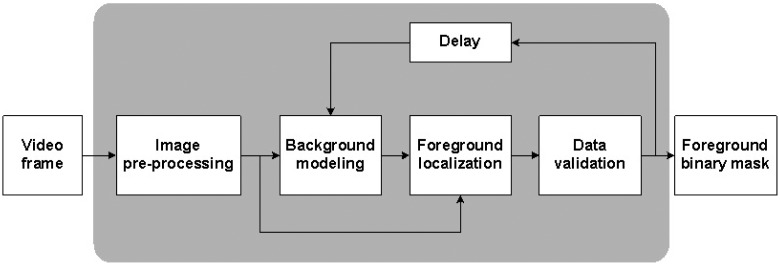
A flowchart of the background subtraction process (based on [1]).

Background modeling detects motion on the basis of the background subtraction process (see Figure 3). As a result of this process, the foreground binary mask is obtained and only objects larger than a pre-specified size are considered as objects of interest (OOIs) for the system. Extracted objects are then tracked under the tracking module. The tracking process is based on the estimation of an object's positions in the subsequent frames. These positions create an object's trajectory. Multiple objects are additionally labeled to maintain the continuity of tracking. Trajectories can be compared in order to recognize potentially dangerous behaviors. The artifacts removal module aims to remove redundant areas from the foreground binary mask, *i.e.*, regions which were mistakenly included in the foreground image. The object classification module enables simple classification of objects on the basis of their parameters or comparison with pre-defined templates. The event detection module focuses on detecting object changes, especially changes in shape and motion. Depending on the user's safety rules or the system's operational status, the system can react to sudden movement, shape change or the lack of movement. System response module determines the system's reaction to the detected event according to the rules set by the user.

## 3. Key Methods and Algorithms Employed in the System for Video Processing Model

Each system module is associated with different actions and each scene environment implies different system working conditions. In result, the system requires adaptation of various methods, algorithms and parameters to be able to operate appropriately in various situations. The “SmartMonitor” system focuses mainly on human silhouette analysis, therefore the most important issues are to build a good background model, extract foreground objects without artifacts, track and classify them if necessary. The algorithms that realize these tasks should be real-time solutions. An extensive body of literature has emerged in the area of video content analysis and machine vision algorithms for recognition, identification and classification of moving objects. This gave a wide selection of proven and reliable algorithms. Many of them have been experimentally tested and some were ultimately chosen and adopted for the system.

The video processing model consists of several steps which are embedded into system modules. The most important steps are associated with the first system module—background modeling—as it influences the subsequent ones. Motion detection in video sequences is based mainly on the background subtraction process which results in foreground mask extraction. Objects appearing in the foreground area are defined as objects of interest for the system if they are coherent and larger than a specified size. Smaller groups of pixels or single isolated pixels are considered as artifacts (false detections), even if they refer to the actual moving object, the object may be located too far from the camera and information about it is insufficient.

Background subtraction is a segmentation technique, hence it has to be resistant to changing weather conditions, able to instantly react to environmental changes occurring in the scene, and detect all foreground objects. Background models can be divided into three categories—static, averaged in time and adaptive. Static background models are simple because the background image does not change over the time. Therefore, foreground extraction involves the subtraction of the same background image from every processed frame [24]. The main drawback of this approach is insensitivity to the variability of real scenes which results in too large size of the extracted foregrounds. This problem is partially solved by the background model averaged in time. A background image is created on the basis of an averaged group of frames and updated periodically at a fixed time [25]. The third background subtraction approach is based on background modeling using Gaussian Mixture Models (GMM) which was proposed by Stauffer and Grimson in [26] and can use both grayscale and color images. GMM models each pixel as a mixture of Gaussian distributions and the background image is updated with every processed frame. In result, it takes into account slow variations in lighting, repetitive background motion and long-term changes in the scene [26].

Based on [26,27], the GMM can be defined as follows: let us assume that an input for the GMM algorithm is the video sequence denoted as *X*_1_, …, *X_t_*, where *X* is a single frame and *t* is a frame index. *M* × *N* × *K* corresponds to frame size, where *M* and *N* are spatial resolutions and *K* is the number of the component of RGB color space. Then the probability *P* of an occurrence of a particular pixel *x_t_*(*m*, *n*) value can be defined using *G* Gaussian distributions and the following formula:

(1) $$P\left(x_{t}\left(m,n\right)\right)=\overset{G}{\underset{i=1}{\text{∑}}}\omega_{i,t}*\eta \left(x_{t}\left(m,n\right),\mu_{i,t},{\text{∑}}_{i,t}\right)$$

where:

*μ_i,t_* —mean value of the *i*th Gaussian in the mixture at time *t*;
Σ*_i,t_*—covariance matrix of the *i*th Gaussian in the mixture at time *t*;
*ω_i,t_* —estimate of the weight;
*σ*—standard deviation;
*η*—Gaussian probability density function.

The initial background image is iteratively adapted by means of the GMM algorithm. Each iteration includes the analysis of every image from the processed frame and the modification of the background model, which involves calculation of the distance *d* between a new pixel value *x_t_*(*m*, *n*) obtained from *X_t_* frame with the actual model:

(2) $$d=\left|\left(x_{t}\left(m,n\right)-\mu \right)\right|$$

If the distance value *d* > *c* · *σ* (*c* is a constant), a pixel does not belong to the background image. In this case, the least probable distribution is replaced with another distribution which includes mean value equal to a current pixel value. Otherwise, if *d* < *c* · *σ*, the model is adapted including new values using formulas:

(3) $$\mu_{t}=\left(1-\rho \right)\mu_{t-1}+\rho x_{t}\left(m,n\right)$$

(4) $$\sigma_{t}^{2}=\left(1-\rho \right)\sigma_{t-1}^{2}+\rho {\left(x_{t}\left(m,n\right)-\mu_{t}\right)}^{T}\left(x_{t}\left(m,n\right)-\mu_{t}\right)$$

where *ρ* = *αη* (*x_t_* (*m*, *n*) |*μ_t_*_−1_, *σ_t_*_−1_) and *α* is a learning constant. Next, estimates of the weight in particular distributions are modified:

$$\omega_{t}=\{\begin{array}{ll} \left(1-\alpha \right)\omega_{t-1}+\alpha & \text{if a new value is mached}\\ \left(1-\alpha \right)\omega_{t-1} & \text{otherwise} \end{array}$$

The modeled distributions are sorted in descending order of the *ω/σ* ratio values and put on a list. The distributions with the highest probability have a high weight and low standard deviation, and are placed at the top of the list. The distributions which are at the bottom of the list are replaced with the new ones. A new scene model is created using *B* first distributions which meet the below condition:

(5) $$B={\text{argmin}}_{b}\left(\overset{b}{\underset{k=1}{\text{∑}}}\omega_{k}>T\right)$$

where *T* is the proportion of the data that should be accounted by the background.

The GMM approach has been also modified and improved by many other researchers. Kaewtrakulpong proposed a reinvestigation of the update equation in order to improve GMM features [28]. Javed focused on performance improvement of the background models in real conditions and proposed the use of color and gradient information to make background subtraction resistant to the sudden changes in lighting [29]. Zivkovic proposed the use of recursive equations to constantly update the parameters of the algorithms and to enable proper selection of components for each pixel [30]. One can ask, why the GMM has to be used for indoors since the majority of elements do not change their positions, like walls, floor or furniture. The answer is simple—there are many smaller objects that can change positions and various lighting conditions are likely to occur, especially sun shining through the window or turning the light on and off. Moreover, there may be constant movements of different origin—small pets or billowing curtains that cannot be taken into consideration by the system, since human silhouettes are of main interest. In the case of frequently changing scenes, the monitored region can additionally be limited to cover only the area in which there is a high probability of an OOI occurrence.

The process of object tracking is defined as determination of an object's position in the subsequent frames [31]. This process consists of several main steps. An initially empty list of objects is created. Currently detected foreground regions are matched to the already tracked objects based on the predicted positions. For each successfully matched object, an algorithm updates its trajectory and predicts its future position. If there are foreground regions not having a matched tracked object, the algorithm creates a new object and adds it to the list. Objects that leave the currently processed frame are removed from the list. Object labeling helps to keep the continuity of tracking especially in case of object occlusion while multiple objects are tracked. Many algorithms for movement tracking assume smooth movement of objects without sudden and unexpected changes in their trajectory or speed [32,33]. Such an assumption simplifies this task, however it decreases the robustness of the algorithm to noise. The assumption of the linearity of the object movement does not work for objects that have stopped or drastically changed their directory. Other problems may arise due to the appearance of objects that move too fast in comparison with the recorded and analyzed number of frames in a given unit of time.

The most important tracking algorithms considered for the application in the “SmartMonitor” system were the Mean-Shift algorithm and Kalman filter. The Mean-Shift is an iterative, appearance-based method which uses features defined by histograms, for instance detected edges or color based on the HSV (Hue-Saturation-Value) color model. The algorithm starts with the selection of a template, *i.e.*, the region that contains a tracked object or a part of it. Then the middle pixel (initial estimate *x*) from the template is selected and a particular feature of a template is calculated. A kernel function *K*(*x_i_* − *x*) is defined. It determines the weight of nearby points used to re-estimate the mean. Function *K* is modeled using Gaussian distributions:

(6) $$K\left(x_{i}-x\right)=\exp^{\left\|x_{i}-x^{2}\right\|}$$

The weighted probability density distribution defined by *K* is calculated using a function *m*(*x*) given by the following formula:

(7) $$m(x)=\frac{\underset{x_{i}\in N(x)}{\text{∑}}K\left(x_{i}-x\right)x_{i}}{\underset{x_{i}\in N(x)}{\text{∑}}K\left(x_{i}-x\right)}$$

where *N*(*x*) is the neighborhood of *x* and *K*(*x*) ≠ 0. The weighted value of *m*(*x*) is assigned to *x* and the algorithm repeats this until convergence is reached [32,34].

In turn, the Kalman filter uses a set of mathematical equations that implement a predictor-corrector type estimator to estimate future positions of each tracked object. The algorithm predicts future values based on noise measurements, corrects the last prediction based on current measurements and updates the estimator state. Because the Kalman filter works well only for linear systems, it requires the simplification of object movement and assuming that objects cannot suddenly change their speed or direction [33]. While in the “SmartMonitor” system focus is placed on event detection, such as faints or falls, the application of Mean-Shift tracking has been selected for the system. Compared to the Kalman filter it preserved the continuity of object tracking even in case when an object changed its movement direction or speed.

The occurrence of false detections is associated with the environment present in the scene and the color model chosen for background modeling, especially when grayscale images are used. The authors of [29] distinguished four types of false detections: sudden illumination changes, background movements, background initialization with foreground objects and moving object shadows. Sudden illumination change can be caused by sunrays passing through the window, a camera flash or turning the light on and off. It increases the difference between the model and the current frame, because the background model is not able to adapt to the changes in a short time and, therefore, large areas of false detections are visible on the background image. Background movement is the relocation of part of the background resulting in changes within newly acquired position and the previous one, and the new region is mistakenly accounted for as a foreground. As a result, the foreground image becomes very noisy. The shadow of a moving object is a rather coherent region that is connected to the actual OOI and moves together with it. Background model initialization in the presence of moving objects causes that these objects are incorporated into the background and occlude part of it.

Each false detection enlarges the overall area of the foreground region and can have a size that varies from single isolated pixels to large coherent groups of pixels connected with an actual OOI. The process of artifacts removal is important for the object analysis, classification and identification based on shape features. As regards the drawbacks and limitations of the GMM algorithm and the types of false detections, the artifact removal process, which uses the foreground binary mask as input has been developed. Single pixels or small groups of several pixels scattered randomly on the foreground image plane can be eliminated using morphological operations, such as erosion and dilation [27]. Particular attention was paid to shadow elimination, because it can significantly increase the area of the foreground object and makes it difficult to separate an actual OOI from its shadow area. The authors of [28] described a method for shadow detection based on the color model. The proposed algorithm is capable of separating information about chrominance and intensity of each pixel. Localization of shadows is performed through the comparison of pixels not belonging to the background with pixels in the background model. If the difference between the pixel intensity and chrominance falls within a pre-defined range, an analyzed pixel is considered as belonging to the shadow area [28]. As stated in [35], a moving shadow causes minor changes in chromaticity. For this reason, many background modeling methods use normalized color which is less sensitive to minor illumination changes caused by shadows than the RGB model [35].

Taking advantage of chrominance information, a method proposed in [36] was used for shadow elimination. It is based on the fact that shadow darkens the regions, while the chrominance of the shaded areas does not vary from the chrominance of the background areas. This method requires the preparation of two background models—one based on the luminance and the other on chromaticity (H component of the HSV color model). In effect the shadow is eliminated by the multiplication of two foreground images using an entrywise product. Moreover, in the final step of the artifacts removal process, objects smaller than a pre-defined size are ignored. The remaining objects are subjected to further processing. The problem of initial model calibration with foreground objects present in the scene occurs when a model is initialized with the first frame from the video stream. In this case an initial background image has to contain random values and be adapted using a group of frames.

In more complex surveillance situations, object localization and extraction is insufficient and human silhouette detection has to be performed. Here the difficulty lies in the need of distinguishing people from other moving objects. Human silhouettes can be detected using the Histogram of Oriented Gradients (HOG) descriptor, which detects objects using predefined patterns and feature vectors. The HOG representation is based on the dominant edge orientations and is derived by calculating the frequency of directional gradients for each of the image blocks. The HOG representation is invariant to translation within the image plane—this means that in the case of an object's position change its directional gradients and histogram remain the same. The HOG algorithm starts with a gamma and color normalization of an image, and subsequently calculates oriented gradients using various directional filters. It detects vertical edges using a filter mask in the form of [−1, 0, 1], and for horizontal edges it uses [1, 0, −1]′. The length of the gradient vector is calculated by means of the formula:

(8) $$\left|G\right|=\sqrt{I_{x}^{2}+I_{y}^{2}}$$

where *I_x_* is a matrix representing an image with the detected vertical edges, and *I_y_* analogously for horizontal edges. A gradient orientation of a grayscale image is derived as follows:

(9) $$\theta =\arctan\frac{I_{y}}{I_{x}}$$

Afterwards, the algorithm divides an image into cells and calculates frequencies of oriented gradients for each cell—the frequencies are later presented on histograms. Cells are grouped into larger blocks that overlap each other and are separately normalized. A block can be square or rectangular, or located in the polar-logarithmic coordinate system. Oriented histograms of all of the cells are concatenated in order to obtain the final HOG descriptor representation [37]. Having divided the image into blocks, the process of object extraction can be performed. It consists of several steps which aim to: determine the template, select the frame size that matches the template, scan the input image and match the template with each block using descriptors, and ultimately produce the matching result and verify it using, e.g., specified threshold values. The result has the form of a greyscale image, in which the darker the region, the greater the similarity to the template.

The second classifier uses Haar-like features [38], as well as the AdaBoost machine learning technique which selects the most appropriate features and correct threshold values. During classification, a cascade built of the Haar-like features is used. It is designed in such a way as to calculate subsequent features of an object only if the answer of the previous feature matches the learned value. Moreover, negative objects are rejected at the earliest stage of recognition [39].

In this section the most important methods and algorithms selected for the “SmartMonitor” system have been briefly described. They were combined into a video processing model to provide reliable and efficient solution consisting of background modeling, foreground extraction, artifacts removal, tracking and object classification. These tasks are realized by the background modeling module, tracking module, artifacts removal module and object classification module. Two other modules are responsible for event detection and system response and they are explained in the fourth section, with a special focus on the feature enabling supervision over ill persons.

## 4. An Analysis of Moving Objects for Faint/Fall Detection

Due to the fact that this paper focuses on the supervision over ill persons, this section deals with the analysis of human silhouettes moving indoors for events detection, particularly faints or falls. In the fall detection scenario we employ a mixture of three commonly used measures, namely: bounding box representing the person [40–42] in the image, its area (denoted in pixels) and a relative speed in the image plane. This method works even if the camera is placed sideways and it does not depend strongly on the camera mounting height (*i.e.*, [43,44]). In contrary to Lee and Mihailidis [43] we detect falls without setting special thresholds for usual inactivity zones like the bed or the sofa. It seems also not necessary to calculate more detailed silhouettes, like in work of Nait-Charif and McKenna [44]. According to our observations, it is also not justified to employ more sophisticated calculations, like large human shape deformation during a fall [45,46] or the three-demensional (3D) vertical velocity [47,48]. The most important is the set of rules that join simple, yet effective geometrical measures. The successful human being identification depends, then, on a rather simple calculations.

The most meaningful feature of an object is its shape, which, in many cases, enables the discrimination of various types of objects. A moving object in a video sequence is in fact a two-dimensional projection of a three-dimensional model. Moreover, cameras capture objects from different angles, what leads to producing many projections of one model. During extensive research, shape analysis based on the template matching approach has been performed. Various shape descriptors have been used to prepare shape representations, for instance simple shape measures and ratios, Fourier-based descriptors, moment-based descriptors or shape descriptors utilizing transformation to the polar coordinate system. However, a detailed shape analysis turned out to be redundant during faint/fall detection. Instead, attention was afforded to the detection and recognition of a change itself and, more specifically, to the change in an object's shape and movement. Moreover, time is also considered as it is crucial for the determination of the duration of a change and the moment in which a change occurred.

Based on the above considerations, two spatiotemporal parameters were determined. Parameter *P* defines the proportion of an object's bounding box, *i.e.*, its aspect ratio (the ratio of width to height). Parameter *K* defines the maximum number of frames, *i.e.*, the time in which a person stays in the protected area or does not move. These parameters are associated with threshold values that determine the moment of an event detection and alarm activation. In other words, when a threshold of a particular parameter is exceeded it means that an event was detected and the alarm is triggered. During the experiments, the suggested threshold values of parameters *P* and *K* were estimated. For parameter *P*, an object's aspect ratio should be between 0.7 and 1.2. In turn, parameter *K* should be between 50 and 150 frames that represent 2–10 s for 15 fps. In order to detect a faint or fall during supervision over an ill person, the system performs several steps. The initial system parameters' values are provided in Table 1. The main and the most important steps of the video processing model for faint/fall detection are listed below:

Step 1. Set initial parameters and threshold values;
Step 2. Open input file (video sequence);
Step 3. Build a background model;
Step 4. Retrieve current video frame;
Step 5. Localize foreground areas in each processed frame;
Step 6. Perform Haar and HOG classification for each detected object;
Step 7. If the classification step gives a positive result, go to Step 8. Otherwise perform Mean-Shift tracking for the recently detected object;
Step 8. Check predefined thresholds for each scenario: check object's position—if the object's position does not change for more than *K* frames and its proportions do not change to exceed *P*, then start the alarm;
Step 9. If the end of the video sequence is not reached, go to step 4. Otherwise terminate the processing.

The problem of faint/fall detection, especially informing about this event and helping a person, can also be supported by home automation functionalities. The main actions which may be performed in response to a faint/fall are: to make a phone call to a responsible person or emergency number, to send images captured by the camera to an email or a mobile phone. Other actions may include, e.g., to start the siren or light signal; turning a light on or rising the blinds to make a room brighter—it would make it easier to stand up or find a thing which was dropped in result of a fall (e.g., glasses); opening a window or turning on ventilation would give a fresh air. However, regardless of whether it was a fall or a fainting of short duration, an immediate reaction is essential. Even a harmless-looking fall can have serious consequences, especially in case of elderly people.

## 5. Experimental Conditions and Results

During the experiments, various video sequences simulating the situation of a fall were used. The position of the human body after the fall in relation to the eye of the camera differs. The video processing model along with the initial system parameters are provided in the fourth section. The input files were retrieved on a sequential read, *i.e.*, at a particular moment only an individual frame was available. It was assumed that the aspect ratio of a standing person's bounding box is close to 0.5. When the aspect ratio rapidly exceeds this threshold and is larger than 0.7, it means that a fall has occurred. The aspect ratio of a bounding box is calculated over several frames, which may represent from 1 to 2 s, depending on the fps. For the second parameter, *P*, a change of the object's centroid position is verified. If the centroid position does not change significantly during a specified period of time, the alarm is triggered. The area of the object's bounding box is also considered during the analysis of the video sequences.

### 5.1. Datasets

**Figure 4.**
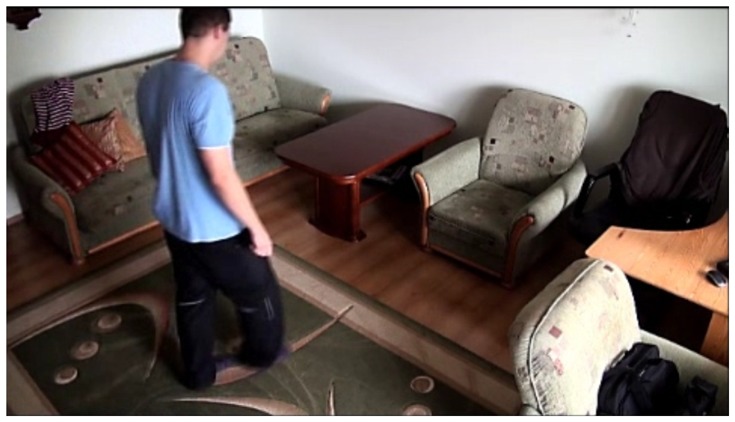
Benchmark video No. 1 (SmartMonitor (“SM”) dataset).

**Figure 5.**
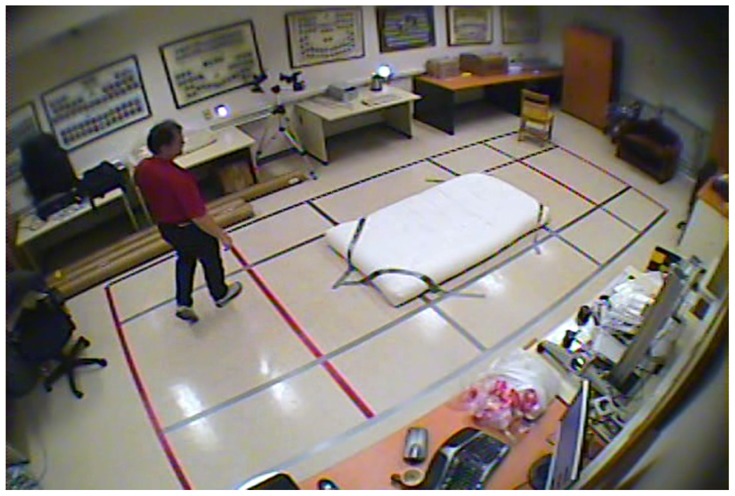
Benchmark video No. 2 (Universite de Montreal (UdM) dataset, scenario 7, camera 3).

**Figure 6.**
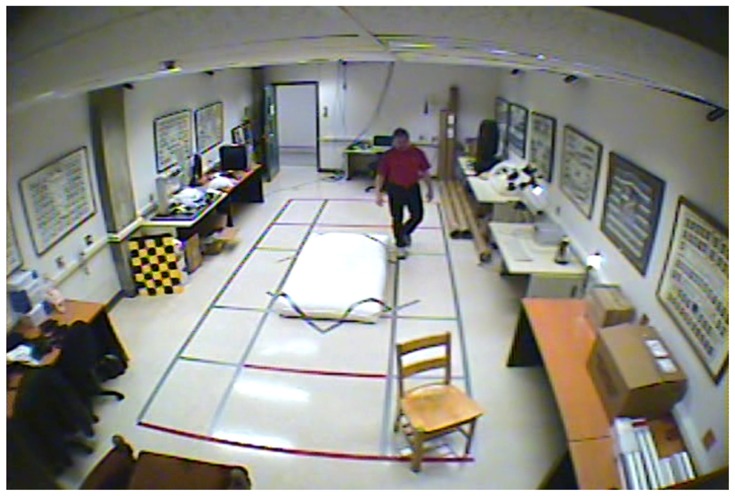
Benchmark video No. 3 (Universite de Montreal (UdM) dataset, scenario 8, camera 5).

In our experiments we used two datasets, each one consisting of several video streams captured in real environment. The first dataset (“SM”—SmartMonitor) is our own benchmark, and consists of four sequences of humans who walk and faint in the small room. The second dataset (“chute”) comes from a technical report coming from Universite de Montreal (UdM), presented in [49]. It consist of multiple sequences, each one presenting different events where the fall is only one of them. This dataset contains 24 sequences recorded with 8 IP video cameras. Sample frames from the benchmark datasets are presented in Figures 4, 5 and 6. The video sequences contain typical difficulties which can lead to segmentation errors like:

- high video compression MPEG4 (Moving Picture Experts Group) which can give artifacts in the image;
- shadows and reflections (causing false detections);
- slightly variable illumination level (influencing the background subtraction);
- disturbing events (entering or leaving field of view).

### 5.2. Parameters Used for Fall Detection

The main measure responsible for fall detection is the aspect ratio and dimensions of a bounding box representing observed person. It is rather easy to discriminate between standing person and a one falling or laying on the floor. In Table 2, one can observe extracted image areas representing observed person. Then, the bounding box is evaluated and its aspect ratio is calculated. It is defined as a proportion of object's height to its width. As it can be seen, the objects with the aspect ratio higher than 0.8–0.9 can be considered as people falling down. When the aspect ratio reaches a value greater than 1.1–1.2, it is possible that a person is lying on the floor.

The other measure that suggests a possible fall event is the area of the bounding box. If it changed, there is a possibility that the object changed its orientation, and together with the above discussed aspect ratio, it points to a fall. The last measure is the speed of object's movement. Although, we do not estimate its direction, we are interested in its general value calculated as a proportion of distance between centers of bounding boxes in successive frames. If the speed is small enough, there is a possibility that a person is lying on the floor.

### 5.3. Experimental Results—Examples of Falls

In this subsection the experimental results based on two exemplary video sequences are discussed and presented in the figures containing a sample frame before an alarm activation, a sample frame after the alarm activation and plots representing object trajectory as XY position of an object's centroid, aspect ratio and area of an object's bounding box in pixels.

**Figure 7.**
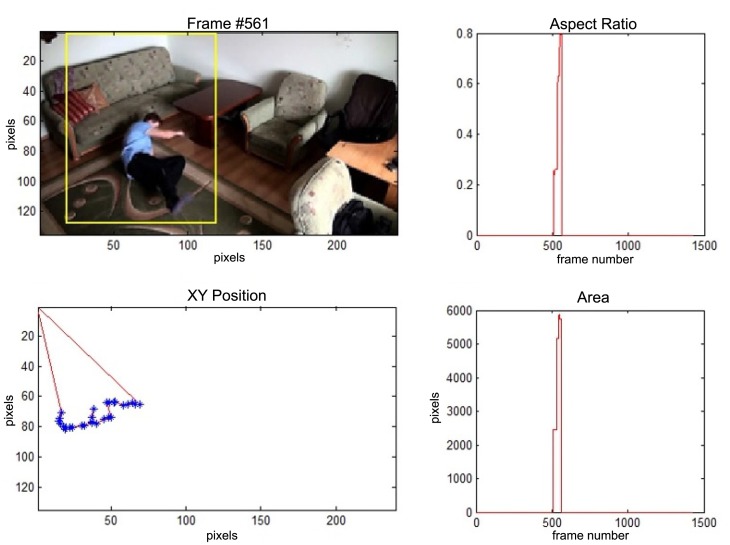
The analysis of an exemplary video sequence—before a fall of a person (“sm_sequence02”).

**Figure 8.**
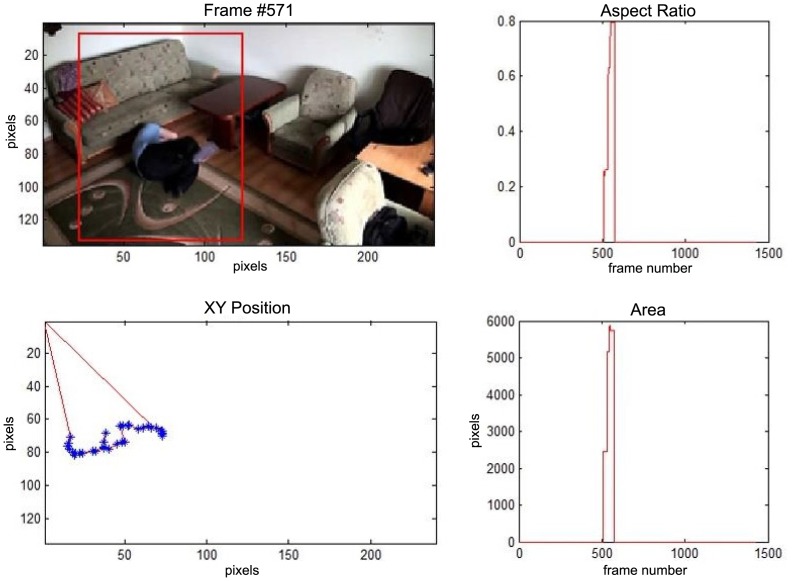
The analysis of an exemplary video sequence—a falling person (“sm_sequence02”).

In the first video sequence a person enters the scene in frame No. 495. The background modeling module detects a moving object and Haar classifier classifies it correctly despite the incompleteness of an object's shape. A person is correctly tracked on the subsequent frames. Around a frame No. 560 a person falls (Figure 7). In frame No. 571, the system marks the person lying on the ground with a red rectangle (Figure 8), because the threshold value of parameter *P* (object's bounding box aspect ratio) is exceeded, and the alarm is triggered.

**Figure 9.**
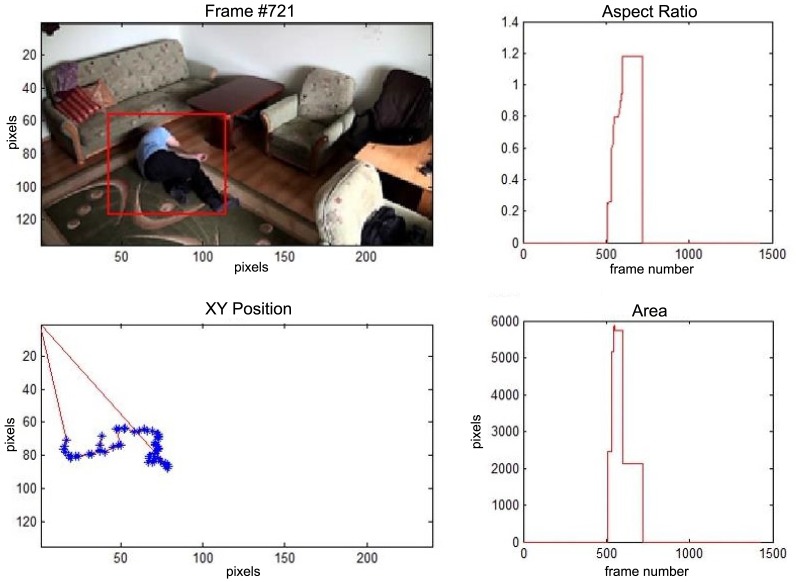
The analysis of an exemplary video sequence—after a fall of a person (“sm_sequence02”).

The change in the object's proportions is smooth which is caused by the specificity of the Mean-Shift algorithm. However, thanks to parameter *K*, which corresponds to the duration of a change, the alarm is triggered properly. A person remains lying still to the end of the sequence and the system constantly informs about the detected event. The exemplary frame with a person lying on the ground is provided in Figure 9. Shape changes are visible on the plots (Figure 8 in accordance to Figure 9).

**Figure 10.**
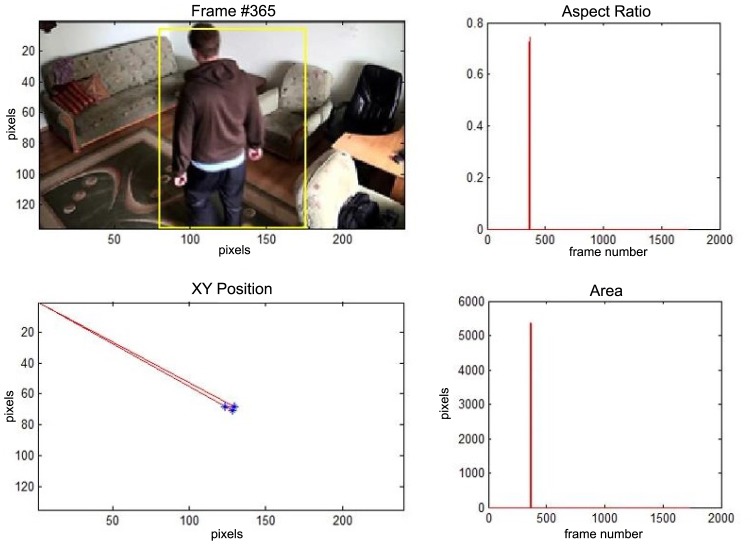
The analysis of an exemplary video sequence—the foreground object is detected, before a fall (“sm_sequence03”).

**Figure 11.**
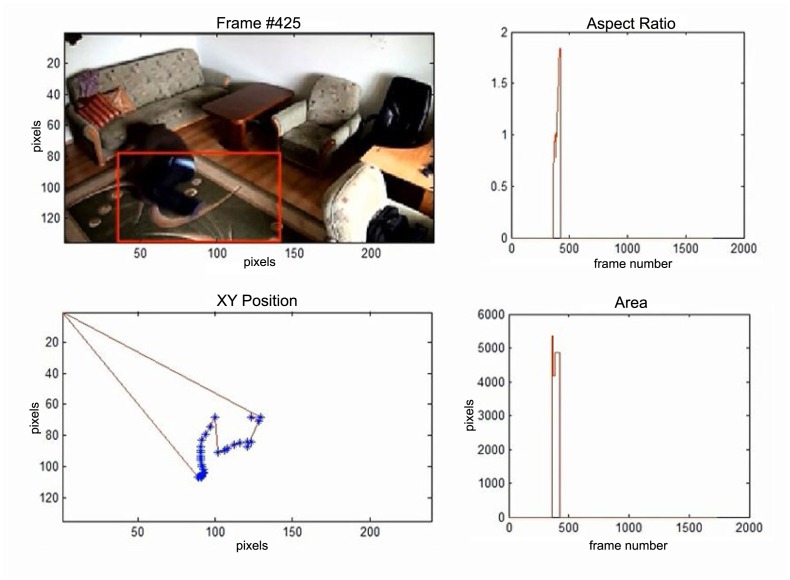
The analysis of another exemplary video sequence—a falling person (“sm_sequence03”).

**Figure 12.**
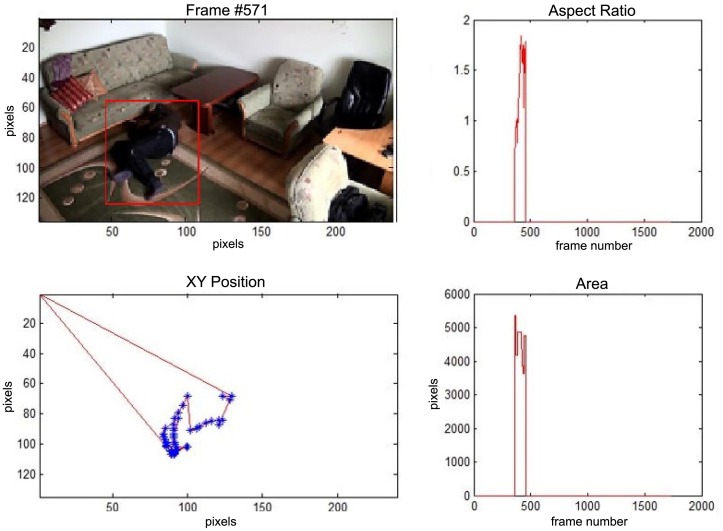
The analysis of another exemplary video sequence—after a fall (“sm_sequence03”).

In the second video sequence, a person appears around frame No. 300 and is correctly detected by the background modeling module. A moving object is correctly classified and tracked despite the fact that only part of it is visible (Figure 10). In frame No. 425, a person falls (Figure 11) and remains lying still until the end of the video sequence is reached. A sample frame containing a person after a fall is depicted in Figure 12.

### 5.4. Experimental Results—The Analysis of Parameters for the Whole Sequences

**Figure 13.**
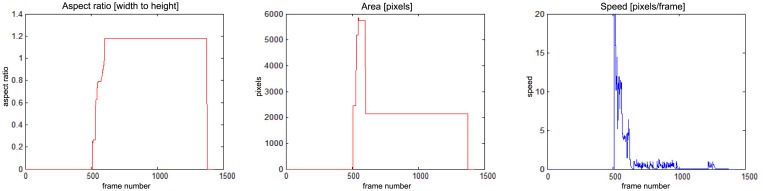
Parameters of moving object evaluated at the action classification stage, for “sm_sequence02”.

The results of fall detection for four sequences are presented in this subsection in a form of charts showing the variability of parameters values. In Figure 13 one can observe that the fall occurs around frame No. 600. The aspect ratio stays constant while the area is smaller than the original object. At the same time the speed of movement is close to zero. If all of these values are constant during a predefined number of frames (time), the alarm is triggered.

**Figure 14.**
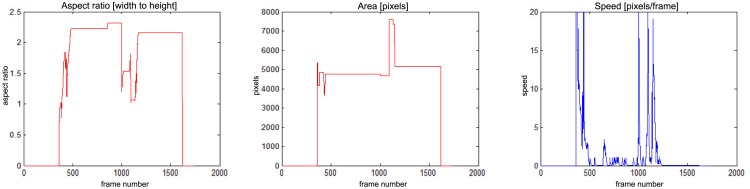
Parameters of moving object evaluated at the action classification stage, for “sm_sequence03”.

In Figure 14 there is a situation, when a person falls around frame No. 500 but moves slightly till frame No. 1200. The alarm is triggered around frame No. 1200, when the speed of movement drops down.

**Figure 15.**
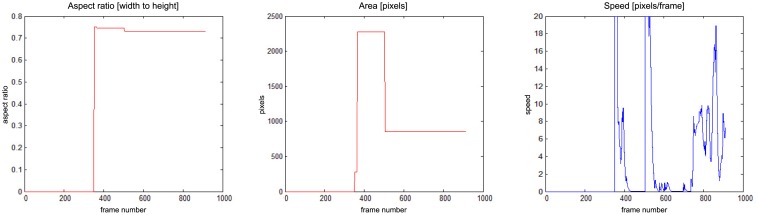
Parameters of moving object evaluated at the action classification stage, for “chute07_cam3”.

In Figure 15 a person enters the field of view around frame No. 350 and moves around till frame No. 500, when a fall is detected. The alarm is triggered around frame No. 550, when the speed of movement drops down. The person moves slightly after that.

**Figure 16.**
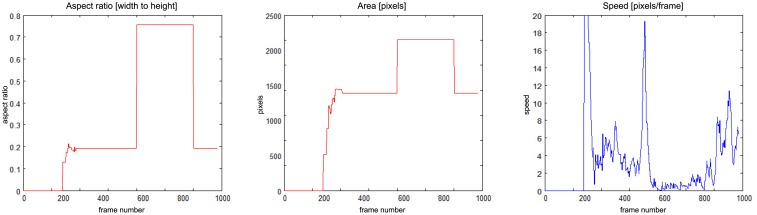
Parameters of moving object evaluated at the action classification stage, for “chute08_cam5”.

There is a situation in Figure 16 when a person enters the field of view around frame No. 140 and moves around till frame No. 400, when a fall is detected. The alarm is triggered around frame No. 400, when the speed of movement drops down. The person moves slightly after that, and around frame No. 600 gets up and moves normally.

### 5.5. Performance Evaluation

Proposed solution works as a prototype system programmed in a heterogeneous environment. The framework is developed in Matlab, while the specific functions—like background model based on Gaussian Mixture Models, image pre-processing (filtering), noise removal, object labeling and object classifier—work as pre-compiled mex-files programmed in C++ and OpenCV Library. In order to estimate the computational overhead, we performed several test runs on video streams in different spatial resolutions. The computer environment consisted of a PC-based notebook equipped with i7 2-core processor, 8 GB of RAM working under 64-bit Windows 7 Professional. The results are presented in Table 3. The base resolution was 720 × 360 pixels and it was downscaled by factors 0.5 and 0.25. As it can be seen, the classifier uses the most of the processing time. It is also interesting that its overhead depends strongly on the number of humans tracked in the video stream. In the idle mode, the processing is not very time consuming. The analysis of these results shows that it is possible to develop such a system for rather low-end computers. Its performance is estimated to 2–5 frames per second, which is sufficient for safe operation.

## 6. Conclusions

The “SmartMonitor” is a solution that takes advantage of video surveillance, alarm and home automation systems and combines them into one multifunctional system. It is based on video content analysis algorithms and aims to protect individual users and their properties in small areas. Another advantage of the system is its customizability and diversity of applications. “SmartMonitor” is also an autonomous solution because human interaction is required only for the system calibration, not during its operation.

In the paper, a general overview of the “SmartMonitor” system was presented. Attention has been focused on how the system operates in supervising ill persons and in the situation of event detection, particularly a fall. This task requires the analysis of an object's shape, mainly how it changes over time. Therefore, two parameters associated with event detection have been established—parameter *P*, which defines an object's bounding box aspect ratio, and parameter *K* associated with the duration of an object's change. The threshold values of these parameters determine the moment in which the alarm is triggered and system response is activated. Experimental results have confirmed the effectiveness of the approach. The developed video processing model appropriately detected, tracked and classified moving objects. The paper also described the logical and physical architecture of the system.

## Figures and Tables

**Table 1.** Initial system parameters. HOG: Histogram of Oriented Gradients.

**Parameter**	**Value**
Frame spatial resolution	640 × 360 pixels
Scaling factor	0.5—320 × 180 pixel frames are processed
Learning mode for background model	40 frames without any movements are used for learning (the model calibrates within 2–3 s)
Morphological operations	Median filter and double erosion using foreground binary mask
The size of the area subjected to the further classification	Minimum 60 pixels
Proportions of object's bounding box that is classified as a human	2:1 (height to width)
Minimum object's size for the Haar classifier	24 × 12 pixels
Minimum object's size for the HOG classifier	96 × 48 pixels
The area on which the tracked object is searched for using the Mean-Shift algorithm	Half of the minimum object's size (width, height)

**Table 2.** Aspect ratios of bounding boxes created for different human silhouettes detected in the exemplary video streams.

seq. 1	0.43	0.45	1.43	1.61	
	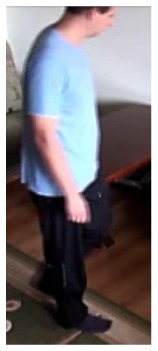	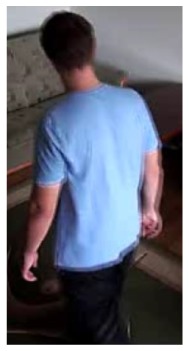	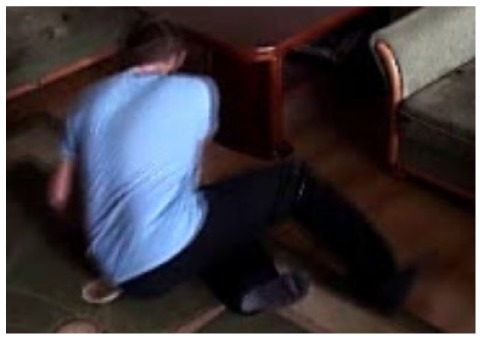	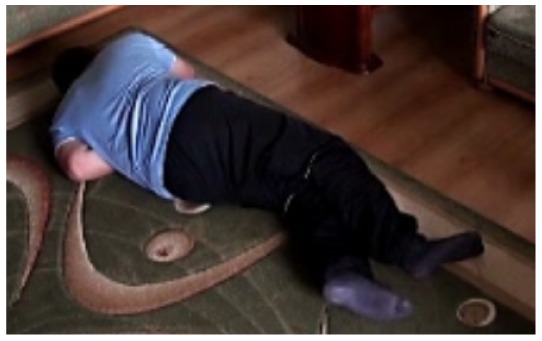	
seq. 2	0.48	0.66	1.04	1.39	1.30
	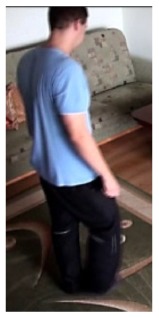	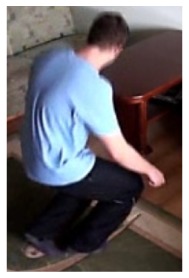	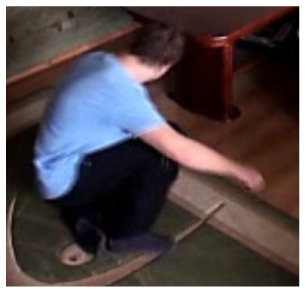	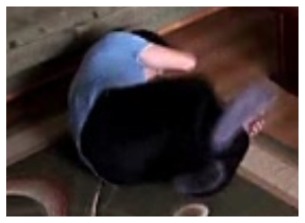	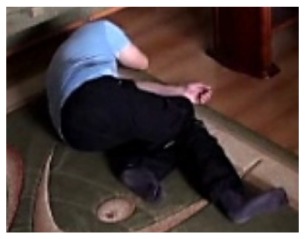
seq. 3	0.52	0.51	0.87	1.32	0.91
	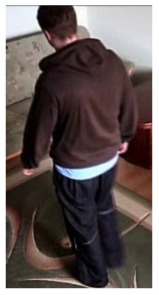	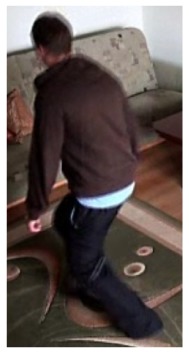	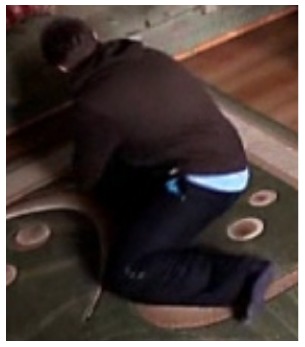	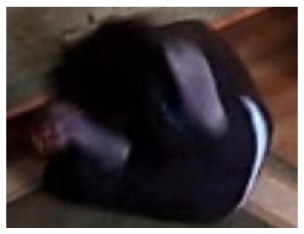	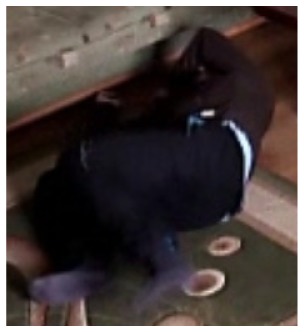
seq. 4	0.46	0.75	1.46	1.45	
	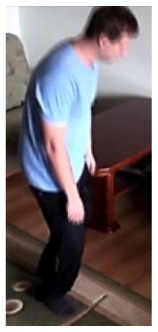	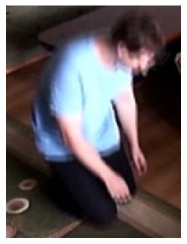	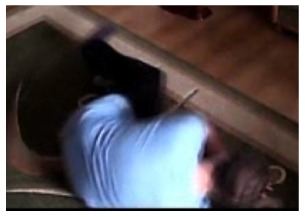	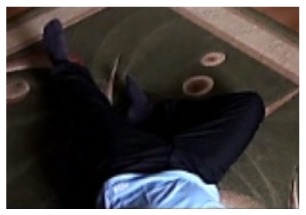	
seq. 5	0.62	0.53	0.84	3.12	1.22
	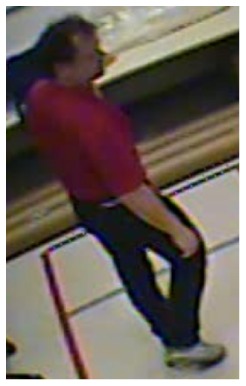	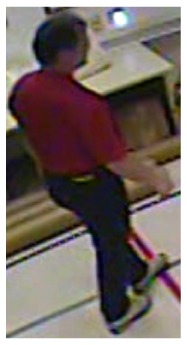	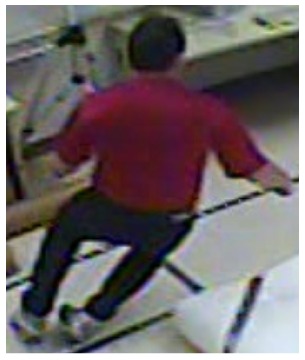	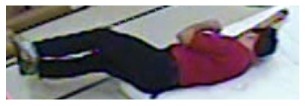	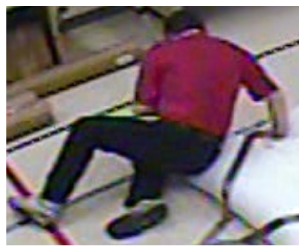
seq. 6	0.50	0.48	0.63	2.28	1.92
	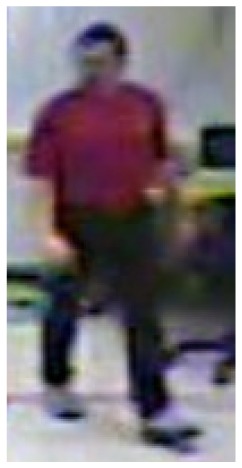	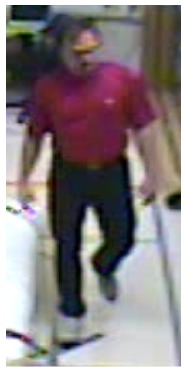	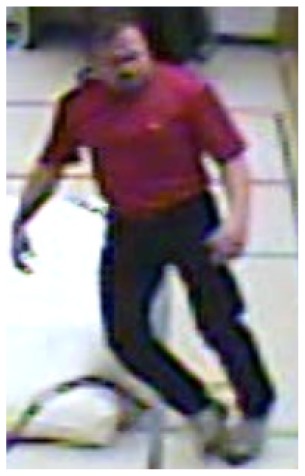	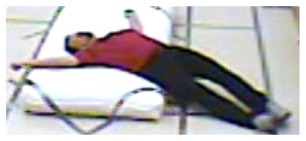	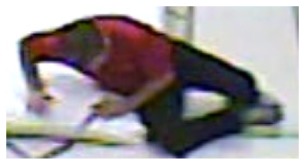

**Table 3.** Performance evaluation.

**Stage of processing**	**Scale 1.0**	**Scale 0.5**	**Scale 0.25**
Background model	210 ms	50 ms	12 ms
Image Processing	110 ms	60 ms	35 ms
Classifier (mean)	640 ms	460 ms	200 ms
Classifier (idle mode)	20 ms	20 ms	10 ms
Classifier (per 1 human/object)	230 ms	300 ms	110 ms

## References

[1] Frejlichowski D., Forczmański P., Nowosielski A., Gościewska K., Hofman R., Bolc L., Wojciechowski K., Tadeusiewicz R., Chmielewski L.J. (2012). SmartMonitor: An approach to simple, intelligent and affordable visual surveillance system. Lecture Notes in Computer Science.

[2] Frejlichowski D., Gościewska K., Forczmański P., Nowosielski A., Hofman R. (2012). SmartMonitor: Recent progress in the development of an innovative visual surveillance system. J. Theor. Appl. Comput. Sci..

[3] Frejlichowski D., Gościewska K., Forczmański P., Nowosielski A., Hofman R., Burduk R., Jackowski K., Kurzyński M., Woźniak M., Żołnierek A. (2013). Extraction of the foreground regions by means of the adaptive background modelling based on various colour components for a visual surveillance system. Advances in Intelligent Systems and Computing.

[4] Frejlichowski D., Gościewska K., Forczmański P., Nowosielski A., Hofman R., Saeed K., Chaki R., Cortesi A., Wierzchoń S. (2013). The removal of false detections from foreground regions extracted using adaptive background modelling for a visual surveillance system. Lecture Notes in Computer Science.

[5] Gil G.B., Bustamante A.L., Berlanga A., Molina J.M. (2012). ContectCare: Autonomus video surveillance system using multi-camera and smartphones. Manag. Intell. Syst..

[6] Nourizadeh S., Deroussent C. Maisons vill'age: Smart use of home automation for healthy aging.

[7] Selim B., Iraqi Y., Choi H.-J. A multi-sensor surveillance system for elderly care.

[8] Lee W., Lee G., Ban S.-W., Jung I., Lee M., Lu B.-L., Zhang L., Kwok J. (2011). Intelligent Video Surveillance System Using Dynamic Saliency Map and Boosted Gaussian Mixture Model. Lecture Notes in Computer Science.

[9] Meinel L., Findeisen M., Hes M., Apitzsch A., Hirtz G. Automated real-time surveillance for ambient assisted living using an omnidirectional camera.

[10] Ge R., Shan Z., Kou H. An intelligent surveillance system based on motion detection.

[11] Wu H., Liu N., Luo X., Su J., Chen L. (2014). Real-time background subtraction-based video surveillance of people by integrating local texture patterns. Signal Image Video Process..

[12] Filonenko A., Jo K.-H. Visual surveillance with sensor network for accident detection.

[13] Castro J.L., Delgado M., Medina J., Ruiz-Lozano M.D. (2011). Intelligent surveillance system with integration of heterogeneous information for intrusion detection. Expert Syst. Appl..

[14] Liu S., Wang P., Wang C., Chang Q. Intelligent security system based on wireless sensor network technology.

[15] Liao Y., Su K. (2011). Multi-robot based intelligent security system. Artif. Life Robot..

[16] Su K.-L., Chia S.-H., Shiau S.-V., Guo J.-H. (2009). Developing a module based security system for intelligent home. Artif. Life Robot..

[17] Wang X. (2013). Intelligent multi-camera video surveillance: A review. Pattern Recognit. Lett..

[18] Bosch Webpage. http://us.boschsecurity.com/us_product/03_solutions_2/solutions.

[19] Bosch IVA Product Data Sheet. http://st-nso-us.resource.bosch.com/media/en/us_product_test/04_customer_service_1/02_contact_8/doc_25/ds_data_sheet_enus_11858668555_iva.pdf.

[20] AgentVI Webpage. http://www.agentvi.com/61-Products-62-Vi_System.

[21] ADT Webpage. http://www.adt.com/video-surveillance.

[22] Vivint Webpage. http://www.vivint.com/en/solutions/.

[23] BRS Labs Webpage. http://www.brslabs.com/.

[24] Gurwicz Y., Yehezkel R., Lachover B. (2011). Multiclass object classification for real-time video surveillance systems. Pattern Recognit. Lett..

[25] Frejlichowski D. Automatic localisation of moving vehicles in image sequences using morphological opertions.

[26] Stauffer C., Grimson W.E.L. Adaptive background mixture models for real-time tracking.

[27] Cheung S.C.S., Kamath C. Robust techniques for background subtraction in urban traffic video.

[28] Kaewtrakulpong P., Bowden R., Remagnino P., Jones G.A., Paragios N., Regazzoni C.S. (2002). An improved adaptive background mixture model for realtime tracking with shadow detection. Video Based Surveillance Systems: Computer Vision and Distributed Processing.

[29] Javed O., Shafique K., Shah M. A hierarchical approach to robust background subtraction using color and gradient information.

[30] Zivkovic Z. Improved adaptive Gaussian mixture model for background subtraction.

[31] Yilmaz A., Javed O., Shah M. (2006). Object tracking: A survey. ACM Comput. Surv..

[32] Comaniciu D., Meer P. (2002). Mean Shift: A robust approach toward feature space analysis. IEEE Trans. Pattern Anal. Mach. Intell..

[33] Welch G., Bishop G. (2006). An Introduction to the Kalman Filter.

[34] Fukunaga K., Hostetler L. (1975). The estimation of the gradient of a density function, with applications in pattern recognition. IEEE Trans. Inf. Theory.

[35] Wang H., Suter D. A re-evaluation of mixture of gaussian background modeling (video signal processing applications).

[36] Forczmański P., Seweryn M., Bolc L., Tadeusiewicz R., Chmielewski L.J., Wojciechowski K. (2010). Surveillance video stream analysis using adaptive background model and object recognition. Lecture Notes in Computer Science.

[37] Dalal N., Triggs B. Histograms of oriented gradients for human detection.

[38] Viola P., Jones M. Rapid Object Detection Using a Boosted Cascade of Simple Features.

[39] Avidan S. (2005). Ensemble tracking. IEEE Trans. Pattern Anal. Mach. Intell..

[40] Toreyin B., Dedeoglu Y., Cetin A. HMM based falling person detection using both audio and video.

[41] Anderson D., Keller J., Skubic M., Chen X., He Z. Recognizing falls from silhouettes.

[42] Tao J., Turjo M., Wong M.-F., Wang M., Tan Y.-P. Fall incidents detection for intelligent video surveillance.

[43] Lee T., Mihailidis A. (2005). An intelligent emergency response system: Preliminary development and testing of automated fall detection. J. Telemed. Telecare.

[44] Nait-Charif H., McKenna S. Activity summarisation and fall detection in a supportive home environment.

[45] Rougier C., Meunier J., St-Arnaud A., Rousseau J. Procrustes shape analysis for fall detection.

[46] Rougier C., Meunier J., St-Arnaud A., Rousseau J. Fall Detection from Human Shape and Motion History Using Video Surveillance.

[47] Wu G. (2000). Distinguishing fall activities from normal activities by velocity characteristics. J. Biomech..

[48] Rougier C., Meunier J., St-Arnaud A., Rousseau J. Monocular 3d head tracking to detect falls of elderly people.

[49] Auvinet E., Rougier C., Meunier J., St-Arnaud A., Rousseau J. (2010). Multiple Cameras Fall Dataset.

